# Case Report: Allogeneic Stem Cell Transplantation Following Induction With CPX-351 in Patients With Acute Myeloid Leukemia Is Feasible

**DOI:** 10.3389/fonc.2020.01746

**Published:** 2020-09-16

**Authors:** Vladan Vucinic, Madlen Jentzsch, Sebastian Schwind, Enrica Bach, Sabine Leiblein, Yvonne Remane, Susanne Rieprecht, Sandra Otto, Anne-Sophie Kubasch, Gerhard Behre, Michael Cross, Uwe Platzbecker, Georg-Nikolaus Franke

**Affiliations:** ^1^University of Leipzig Medical Center, Clinic and Policlinic for Hematology and Celltherapy, Leipzig, Germany; ^2^University of Leipzig Medical Center, Pharmacy, Leipzig, Germany

**Keywords:** CPX-351, AML-MRC, therapy-related acute myeloid leukemia, induction therapy, hematopoeietic stem cell transplantation, prognosis

## Abstract

Acute myeloid leukemia with myelodysplasia-related changes (AML-MRC) and treatment-related acute myeloid leukemia (tAML) after chemotherapy or radiation therapy for other neoplasms are associated with poor outcomes. CPX-351, a dual-drug liposomal encapsulation of daunorubicin and cytarabine, has been shown to improve outcomes in AML-MRC and tAML compared with standard 7+3 regimens. Here we report the cases of four consecutive patients with AML-MRC or tAML who received CPX-351 as outpatient induction therapy immediately followed by allogeneic hematopoietic stem cell transplantation (allo-HSCT). Two patients received allo-HSCT in remission (one in complete remission and one in partial remission) and two patients received allo-HSCT in aplasia (one at 11 days and one at 52 days after the start of induction therapy with CPX-351). With a median follow-up of 188 days after allo-HSCT, all but one patient are alive and two are in remission. Further studies will help define and expand the role of CPX-351 in the treatment of AML-MRC and tAML, especially in patients expected to undergo allo-HSCT.

## Introduction

Acute myeloid leukemia (AML) is a hematologic neoplasm characterized by the clonal expansion and subsequent increase of malignant myeloid precursors in the bone marrow and peripheral blood (1). AML may develop either *de novo* or following an antecedent hematologic disorder, as is the case for AML with myelodysplasia-related changes (AML-MRC) or as a late complication of chemotherapy or ionizing radiation (treatment-related AML [tAML]) (1). AML-MRC and tAML account for approximately one-quarter of all AML cases and occur more frequently in older patients (2). Independent of advanced age, several features are associated with poor outcomes in these patients, including a high incidence of adverse/complex cytogenetics and multidrug resistance (2–6). The consolidation therapy offering the highest chance of sustained complete remission (CR) in AML patients is allogeneic hematopoietic stem cell transplantation (allo-HSCT) (7, 8). Because standard myeloablative conditioning is not feasible for patients with comorbidities or for those of advanced age, reduced intensity conditioning regimens that rely to a larger extent on immunological graft-versus-leukemia effects have been developed to allow allo-HSCT in these patients (9, 10).

CPX-351 (VYXEOS® Jazz Pharmaceuticals, Palo Alto, CA, USA) is a dual-drug liposomal encapsulation of daunorubicin and cytarabine formulated at a fixed synergistic molar ratio of 1:5 (11–14). CPX-351 was approved in 2017 by the US Food and Drug Administration and in 2018 by the European Medical Agency for the treatment of adults with newly diagnosed tAML or AML-MRC (15, 16).

## Patients

The cases presented here include four Caucasian patients (three males, one female) with a history of thyroid cancer, breast cancer, myelodysplastic syndrome (MDS), or chronic lymphocytic leukemia who received CPX-351 induction therapy in the outpatient unit at the University of Leipzig Medical Center following a diagnosis of tAML or AML-MRC. All treatments were performed outside of a clinical trial. All patients were older than 60 years at AML diagnosis. The median bone marrow blast count at diagnosis was 25% (range: 20–48%). Of the four patients, two were classified as being adverse risk and two as intermediate risk according to the European LeukemiaNet 2017 classification system (7). The two patients with antecedent MDS received previous treatment with hypomethylating agents. Detailed patient characteristics are shown in Table 1.

**Table 1:** Patient characteristics.

**Patient sex and age, years**	**Diagnosis**	**Karyotype**	**Mutation status**	**ELN 2017 risk group (7)**	**Interval from CPX-351 to allo-HSCT, days**	**Donor**	**HCT-CI score prior to allo-HSCT**	**DRI-score prior to allo-HSCT**	**Conditioning regimen**	**Immunosuppression**	**Outcome after allo-HSCT**	**Acute GvHD**	**Follow-up time from allo-HSCT, days**
Male, 66	tAML after CLL	Complex (7)	*NPM1* and *CEBPA* wild type; *FLT3*-ITD and *FLT3*-TKD negative	Adverse	52	MUD (male)	0	Very high	Fludarabine 30 mg/m^2^ d −12 to d −9; cytarabine 2,000 mg/m^2^ d −12 to d −9; amsacrine 100 mg/m^2^ d −12 to d −9; cyclophosphamide 60 mg/kg d −5, d −4; ATG 10 mg/kg d −4 to d −2; 4 Gy TBI d −1	CSA d −1 to d +99	Alive, CR	No	287
Male, 61	AML-MRC after MDS	46, XY	*SF3B1* mutated (VAF 21%); wild type for *ASXL1, CEBPA, CBL, DNMT3A, EZH2, FLT3, IDH1, IDH2, JAK2, NPM1, RUNX1, SRSF2, TET2, TP53, U2AF1, ZRSR2*	Intermediate	37	Haplo-identical (daughter)	0	Intermediate	Busulfan 1.6 mg/kg q12 hours d −4, d −3; Fludarabine 30 mg/m^2^ d −6 to d −2	Cyclophosphamide 50 mg/kg d +3, d +4; MMF d +5 to d +28; Tac d +5 ongoing	Alive, CR	No	188
Male, 67	AML-MRC after tMDS and thyroid cancer	Complex (7)	*TP53* mutated (VAF 13%); wild type for *ASXL1, BCOR, BCORL1, CBL, DNMT3A, EZH2, FLT3, GATA2, IDH1, IDH2, JAK2, NPM1, PIGA, RUNX1, SF3B1, SRSF2, U2AF1, ZRSR2*	Intermediate	11	MMUD (male)	>3	High	Busulfan 1.6 mg/kg q12 hours d −4, d −3; fludarabine 30 mg/m^2^ d −6 to d −2	MTX 15 mg/m^2^ d +1 and MTX 10 mg/m^2^ d +3, d +6; CSA d −1 to d +90	Alive, relapse after 90 days	No	157
Female, 68	tAML after breast cancer	46, XX, *t*_(2;11)_, del(7p)	*FLT3*-ITD positive (ratio 0.019); *NPM1* and *CEBPA* wild type; negative for *AML1-ETO, CBFB-MYH11, FLT3-*TKD	Adverse	59	MUD (female)	>3	High	Fludarabine 30 mg/m^2^ d −12 to d −9; cytarabine 2,000 mg/m^2^ d −12 to d −9; amsacrine 100 mg/m^2^ d −12 to d −9; cyclophosphamide 60 mg/kg d −5, d −4; ATG 10 mg/kg d −4 to d −2; 4 Gy TBI d −1	CSA d −1 to d +26	Death on day 26	No	26

*allo-HSCT, allogeneic hematopoietic stem cell transplantation; AML, acute myeloid leukemia; AML-MRC, acute myeloid leukemia with myelodysplasia-related changes; ATG, anti-thymocyte globulin; CLL, chronic lymphocytic leukemia; CR, complete remission; CSA, cyclosporine A; DRI, disease risk index; ELN, European LeukemiaNet; FLAMSA, sequential fludarabine, intermediate-dose cytarabine, amsacrine, total-body irradiation/busulfan, and cyclophosphamide; GvHD, graft-versus-host disease; HCT-CI, hematopoetic cell transplantation—specific comorbidity index; ITD, internal tandem duplication; MDS, myelodysplastic syndrome; MMF, mycophenolate mofetil; MMUD, mismatched unrelated donor; MUD, matched unrelated donor; MTX, methotrexate; sAML, secondary acute myeloid leukemia; Tac, tacrolimus; tAML, therapy-related acute myeloid leukemia; TBI, total-body irradiation; TKD, tyrosine kinase domain; tMDS, therapy-related MDS; VAF, variant allele frequency*.

All patients received induction therapy with CPX-351 (44 mg/m^2^ daunorubicin and 100 mg/m^2^ cytarabine) as a 90-min infusion on days 1, 3, and 5. The day 5 dose was delayed in one patient because of a grade 3 adverse event (skin rash); the day 5 dose could not be administered at all in another patient because of donor availability (conditioning prior to allo-HSCT was required to be initiated on day 5 of induction therapy). Febrile neutropenia occurred in one patient, and one patient with neutropenia developed a dental root inflammation requiring tooth extraction. The bone marrow assessments on day 15 after induction therapy showed a reduction of blast counts to <5% in two patients. One patient showed a reduction of blasts from 20 to 10%, and another patient had no detected change in blast count.

Hematological reconstitution occurred prior to allo-HSCT in two patients. The recovery of white blood cells (WBCs; to >1 Gpt/L) occurred at 21 and 33 days, and recovery of platelets (to >50 Gpt/L) occurred at 30 and 37 days after induction with CPX-351, respectively.

CPX-351 therapy was well-tolerated, with no chemotherapy-related alopecia. No patient showed a >10% decrease in body weight between the start of therapy and allo-HSCT, while one patient gained 1.7 kg and one gained 2.7 kg. Unrelated donors were identified for three of the four patients (two human leukocyte antigen [HLA] matched and 1 HLA-A antigen mismatched). For the remaining patient, a haplo-identical daughter was identified as a graft source.

The hematopoetic cell transplantation–specific comorbidity index (HCT-CI) (17) was determined prior to allo-HSCT in all patients showing high risk in two patients. Disease Risk Index (DRI) (18) showed very high risk in one patient and high risk in two patients.

Overall, two patients underwent allo-HSCT after achieving remission (one patient was in CR and 1 was in PR) and two patients underwent allo-HSCT with aplasia (one in a leukemia-free state and one with no response determined by both bone marrow morphology and flow cytometry).

Allo-HSCT was performed at a median of 44.5 days (range: 11–59) after the start of induction therapy using a median of 7.33 × 10^6^ CD34+ cells/kg body weight (range: 6.4–9.8) following conditioning with busulfan/fludarabine (two patients) or sequential fludarabine, intermediate-dose cytarabine, amsacrine, total-body irradiation/busulfan, and cyclophosphamide (FLAMSA; two patients). One patient conditioned with FLAMSA developed multiple infectious complications in neutropenia, which resulted in septic shock on day 0 with consecutive multiorgan failure and death on day 26 after allo-HSCT. This patient showed WBC reconstitution (WBC > 1 Gpt/L) on day 15 after allo-HSCT, but no platelet recovery. Bone marrow assessment was not performed prior to death. Prior to allo-HSCT this patient was in CR.

Of the remaining three patients, two developed a single episode of febrile neutropenia after allo-HSCT. One of these patients had undergone allo-HSCT while neutropenic at day 11 after the start of induction therapy. All three patients showed WBC reconstitution on a median of day 15 (range: 14–15) after allo-HSCT and platelet reconstitution (to >50 Gpt/L) on days 13, 18, and 29, respectively. All three patients were in CR with 100% donor bone marrow chimerism on day 28. None of these three patients showed signs of acute graft-versus-host disease (GvHD).

Immunosuppressive therapy was discontinued in two patients, 1 after 90 days and 1 after 99 days. One of them relapsed at day 90 after allo-HSCT, and the other is in a persisting CR after conditioning according to the FLAMSA protocol. At 157 days after allo-HSCT, the patient with relapse was alive and receiving treatment with 5-azacitidine combined with donor lymphocyte infusion. The bone marrow smear on day 135 after allo-HSCT showed 7% blasts with 80% total chimerism. One patient in CR is still receiving tacrolimus due to mild chronic cutaneous GvHD on day 188 after allo-HSCT. With a median follow-up of 188 days, three of four patients are alive and two are in persisting CR.

## Discussion

Here we report our experience of allo-HSCT following induction therapy with CPX-351 in four patients with tAML or AML-MRC. Induction therapy with CPX-351 was associated with adverse effects previously described as occurring during induction therapy (i.e., febrile neutropenia and skin rash), and all four patients were able to proceed to allo-HSCT. CPX-351 was administered to all patients in an outpatient setting. No alopecia or significant weight loss occurred, which are relevant issues positively affecting patients' quality of life. Two patients underwent allo-HSCT in aplasia, one after 11 days and one after 52 days. Interestingly, no patient in our cohort developed signs of acute GvHD. One could speculate that the tissue damage caused by induction therapy with CPX-351 was less severe than that with the standard 7+3 induction regimen of cytarabine and daunorubicin, but this remains to be examined in prospective clinical trials.

CPX-351 has been evaluated in a phase 3 study in older patients with tAML and AML-MRC and showed both higher response rates and survival benefit compared with the 7+3 induction regimen irrespective of age, *FLT3* status, or AML subtype (tAML, AML with antecedent MDS or chronic myelomonocytic leukemia [CMML]), and cytogenetic risk classification (15). Furthermore, CPX-351 had a better toxicity profile than the standard chemotherapy. In the CPX-351 cohort, 34% patients underwent allo-HSCT compared with only 25% in the 7+3 cohort. The majority of patients who underwent allo-HSCT in both cohorts were in CR or in CR with incomplete hematologic recovery. An exploratory survival analysis landmarked from the time of allo-HSCT also favored CPX-351, with median overall survival not reached in the CPX-351 cohort vs. 10.25 months in the 7+3 cohort. One explanation for this finding could be the deeper molecular remission in the CPX-451 cohort.

In a recently published *post-hoc* analysis of the phase 3 study, CPX-351 was tested as a consolidation regimen after its use as induction therapy and was proven superior to a 5+2 consolidation regimen of cytarabine and daunorubicin after the 3+7 induction, providing a median overall survival of 25.4 vs. 8.5 months, with an acceptable safety profile (19). CPX-351, as both induction and consolidation therapy, can be administered in the outpatient setting in some cases, thus improving the treated patients' quality of life. The therapy should be administered by an experienced team, with careful monitoring during and after treatment administration.

One other valid outpatient option for elderly comorbid patients is the combination of venetoclax with hypomethylating agents (HMA) or low dose cytosinarabinoside. A Phase Ib trial of a combination of venetoclax with HMA in treatment-naïve elderly patients not eligible for intensive induction therapy showed a CR+ CRi rate of 67% after a median follow-up of 8.9 months (20). Similar data were confirmed outside of clinical trials (21).

Further investigations are necessary to define the role of CPX-351 in consolidation and possibly as salvage therapy.

## Conclusions

This case series reports on four patients with tAML and AML-MRC who received CPX-351 induction therapy followed by allo-HSCT. CPX-351 was well-tolerated, and no patients showed signs of acute GvHD. At the time of writing this report, three of the four patients are still alive and two are in persisting CR. Further studies will help define and expand the role of CPX-351 in the treatment of AML.

## Data Availability Statement

The raw data supporting the conclusions of this article will be made available by the authors, without undue reservation.

## Ethics Statement

Ethical review and approval was not required for the study on human participants in accordance with the local legislation and institutional requirements. The patients/participants provided their written informed consent to participate in this study. Written informed consent was obtained from the individual(s) for the publication of any potentially identifiable images or data included in this article.

## Author Contributions

VV, UP, MJ, SS, and G-NF contributed to the design, analysis of this study, and writing of the manuscript. MJ, G-NF, SS, EB, SL, YR, SR, A-SK, MC, VV, GB, SO, and UP were involved directly or indirectly in the care of patients. MJ, SS, A-SK, EB, and SL were involved in sample procurement. All authors agreed on the final version.

## Conflict of Interest

VV, MJ, SS, GB, G-NF, and UP received Honoraria from Jazz Pharmaceuticals. MJ, SS, VV, G-NF, A-SK, and UP received travel reimbursements by Jazz Pharmaceuticals. The remaining authors declare that the research was conducted in the absence of any commercial or financial relationships that could be construed as a potential conflict of interest.
